# Evaluation of a Peptide Hydrogel as a Chondro-Instructive Three-Dimensional Microenvironment

**DOI:** 10.3390/polym15244630

**Published:** 2023-12-06

**Authors:** Rodrigo Nogoceke, Raphaella Josino, Anny Waloski Robert, Marco Augusto Stimamiglio

**Affiliations:** Stem Cells Basic Biology Laboratory, Instituto Carlos Chagas—ICC-FIOCRUZ/PR, Rua Professor Algacyr Munhoz Mader, 3775, Curitiba 81350-010, Brazil; rodrigo_nogoceke@hotmail.com (R.N.); raphaella.josino@outlook.com (R.J.)

**Keywords:** peptide hydrogels, Puramatrix, adipose-derived stem cells, chondrogenic differentiation

## Abstract

Articular cartilage injuries are inherently irreversible, even with the advancement in current therapeutic options. Alternative approaches, such as the use of mesenchymal stem/stromal cells (MSCs) and tissue engineering techniques, have gained prominence. MSCs represent an ideal source of cells due to their low immunogenicity, paracrine activity, and ability to differentiate. Among biomaterials, self-assembling peptide hydrogels (SAPH) are interesting given their characteristics such as good biocompatibility and tunable properties. Herein we associate human adipose-derived stem cells (hASCs) with a commercial SAPH, Puramatrix™, to evaluate how this three-dimensional microenvironment affects cell behavior and its ability to undergo chondrogenic differentiation. We demonstrate that the Puramatrix™ hydrogel comprises a highly porous matrix permissible for hASC adhesion and in vitro expansion. The morphology and cell growth dynamics of hASCs were affected when cultured on the hydrogel but had minimal alteration in their immunophenotype. Interestingly, hASCs spontaneously formed cell aggregates throughout culturing. Analysis of glycosaminoglycan production and gene expression revealed a noteworthy and donor-dependent trend suggesting that Puramatrix™ hydrogel may have a natural capacity to support the chondrogenic differentiation of hASCs. Altogether, the results provide a more comprehensive understanding of the potential applications and limitations of the Puramatrix™ hydrogel in developing functional cartilage tissue constructs.

## 1. Introduction

Articular cartilage lesions are a consequence of the degradation of cartilaginous tissue in response to various metabolic, genetic, vascular, or traumatic stimuli. Cartilage damage can arise from a single instance of excessive load on the joint or the accumulation of repeated minor episodes [1]. These types of lesions are relatively common, as evidenced by a comprehensive review of 31,516 cases of knee arthropathy involving patients over 20 years old conducted by Curl et al. [2]. Their study revealed that 63% of the knees examined exhibited chondral lesions, while 20% presented osteochondral lesions. Notably, 5% of these cases occurred in patients under the age of 40. Unfortunately, a significant proportion of chondral lesions are inherently irreversible, even with the current therapeutic options available. This limitation arises from the intrinsic characteristics of cartilage, including its lack of vascularization, innervation, lymphatic drainage, and readily available progenitor cells for repair [3].

Among the cartilage regeneration strategies, autologous chondrocyte implantation (ACI) is currently considered the gold standard for treating such lesions. In ACI, chondrocytes are isolated from healthy cartilage regions in the patient, cultured in the laboratory, and subsequently re-implanted into the damaged area, aiming to repopulate the tissue, increasing the production of cartilaginous extracellular matrix (ECM) and promoting tissue regeneration [4]. Although articular chondrocytes can be considered the logical choice of cells, they present certain limitations, such as instability and dedifferentiation during monolayer culture, as well as a requirement for a limited amount of healthy donor tissue [5]. Consequently, alternative cell sources, such as mesenchymal stem/stromal cells (MSCs), have gained significant attention. MSCs can not only differentiate into chondrocytes, promoting the formation of new cartilage, but they also exhibit paracrine effects on resident cells [6]. By releasing bioactive factors, extracellular vesicles, and components of the ECM, they actively modulate the behavior of receptor cells, including chondrocytes, endogenous stem cells, and macrophages, influencing crucial cellular processes such as proliferation, differentiation, migration, macrophage polarization, metabolism, and apoptosis [7]. Therefore, MSCs can effectively regulate the local microenvironment, contributing to the repair and regeneration of damaged cartilage. Considering these promising characteristics, MSCs have already been tested in clinical trials for the treatment of articular lesions, with observed outcomes in terms of improved physical functioning surpassing ACI [8]. However, intra-articular injection of stem cells for cartilage injury treatment presents significant limitations, including the high rate of cell death at the injection site [9] and the extravasation of these cells into locations beyond the joint space, given their tendency to migrate to other organs or tissues [10]. To address these limitations, tissue engineering techniques utilizing three-dimensional scaffolds, particularly hydrogels, have been employed to enhance the delivery and survival of stem cells.

Hydrogels have gained prominence in cartilage tissue engineering due to their resemblance to the native cartilaginous matrix, characterized by a porous structure that enables cellular transport and proliferation [3]. Moreover, hydrogels can be minimally invasively injected into damaged tissue [11]. Self-assembling peptide hydrogels have garnered attention in the field of cartilage regeneration, primarily due to their biocompatibility and tunable properties, mimicking the mechanical characteristics of natural cartilage [12]. One notable peptide hydrogel, RADA-16 (RADARADARADARADA), commercially known as Puramatrix™, holds great promise in the research and treatment of articular lesions. It forms a stable three-dimensional structure with certain permeability, closely resembling the native 3D ECM, facilitating cell adhesion and migration [13]. Hence, it serves as an ideal model for cartilage injury investigations. Liu and colleagues [14] demonstrated that chondrocytes seeded in Puramatrix™ maintained their cartilage phenotype and produced cartilaginous ECM, with type II collagen expression sustained for at least three weeks after seeding [14]. Another study observed that bovine MSCs encapsulated within Puramatrix™ hydrogel led to increased glycosaminoglycan (GAG) production and type II collagen deposition [15]. Additionally, the association between Puramatrix™ coating and quail embryonic neural crest-derived cells resulted in the formation of GAG-rich cartilaginous nodules and significant chondroitin sulfate detection [16].

Despite these and other advances in the development of biomimetic cartilage tissues, there are several challenges regarding the regenerative potential and effectiveness of different approaches. These include (1) the selection of appropriate biomaterials, (2) the utilization of 3D structures, (3) the availability of molecular cues, and (4) the identification of optimal cell types and combinations of cells and growth factors. Addressing these peculiarities is crucial for optimizing cartilage regeneration strategies. Thus, this study investigated the association between human adipose-derived stem cells (hASCs) and the Puramatrix™ hydrogel, evaluating how this 3D microenvironment affects their behavior and ability to undergo chondrogenic differentiation. By doing so, we expect to contribute to the development of improved strategies for cartilage regeneration.

## 2. Materials and Methods

### 2.1. Isolation and Culture of hASCs

This study was performed following the guidelines for research involving human subjects laid out in the Declaration of Helsinki and with donors providing informed consent. It was also reviewed and approved by the Research Ethics Committee of Fundação Oswaldo Cruz, Brazil (approval number CAAE 48374715.8.0000.5248). The hASCs were isolated from the adipose tissue of three healthy female donors (BMI: 22.89 + 0.969; Age: 25.67 + 4.93) who had undergone liposuction surgery following a protocol previously described by Rebelatto et al., 2008. The cells’ identity was later confirmed [17,18] through flow cytometry immunophenotyping and evaluation of adipogenic and osteogenic differentiation potential, according to Dominici and colleagues, 2006. The cells were cultivated in Dulbecco’s modified Eagle’s medium (DMEM) (Gibco Invitrogen^®^, Carlsbad, CA, USA) supplemented with 10% fetal bovine serum (FBS) (Gibco Invitrogen^®^, Carlsbad, CA, USA), 4 mM l-glutamine (Gibco Invitrogen^®^, Carlsbad, CA, USA), 100 U/mL penicillin and 100 µg/mL streptomycin (Sigma-Aldrich, Saint Louis, MO, USA) at 37 °C in a 5% CO_2_ atmosphere. hASCs between passages 4 and 7 were used for the experiments. All experiments were performed under hypoxic conditions (at 37 °C in a 5% CO_2_ and 5% O_2_ atmosphere).

### 2.2. Puramatrix™ Coating Preparation

To evaluate the influence of Puramatrix™ (Corning^®^ PuraMatrix™ Peptide Hydrogel; Sigma-Aldrich, St. Louis, MO, USA) on the hASCs, 0.5% Puramatrix™ (diluted in 20% sucrose solution) was adsorbed to the plate wells before each experiment. Puramatrix™ rapidly undergoes self-assembly when it comes into contact with the cell culture medium during the cell seeding process, creating a thin layer of hydrogel on which the hASCs are cultivated.

### 2.3. Hydrogel Characterization Using Scanning Electron Microscopy

**Figure 1:**
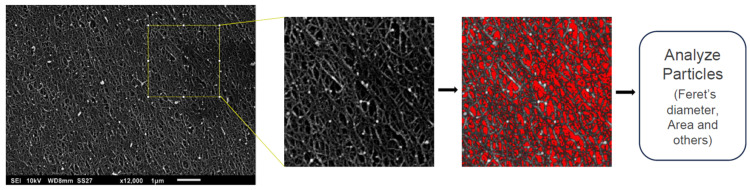
Pore size analysis using ImageJ software. The original image was used to define the scale. Then, a region of the image was selected and the threshold was defined. Finally, the particles were analyzed and information on pore size was obtained (Feret’s diameter and area).

Scanning electron microscopy (SEM) was performed to characterize the Puramatrix™ adsorbed on the cell culture plate as well as the morphology of the cells adhered to the Puramatrix™ coating (PURA). For hydrogel characterization, coated surface samples were analyzed before cell seeding, while for the cell adhesion analysis, cells were cultured on PURA for periods of 5 and 10 days. The samples were washed with PBS and then fixed with 2.5% glutaraldehyde (in 0.1 M sodium cacodylate buffer) for 1 h at room temperature. The post-fixation process was carried out by washing the samples thrice with a 0.1 M sodium cacodylate buffer and incubating the samples in a solution of 1% osmium tetroxide (in 0.1 M sodium cacodylate buffer) for 40 min. Samples were dehydrated using increasing concentrations of ethanol (30–100%), then subjected to critical point drying, coated with gold, and analyzed using a scanning electron microscope (JEOL JSM6010 PLUS-LA, JEOL Ltd., Tokyo, Japan). To estimate the pore size of Puramatrix™ coating, we used Image J 1.54g software. Briefly, after setting the brightness and contrast, we defined the image scale (nm). Afterward, a region of the image was selected and cropped, and then the threshold was defined (to select the pores). We then applied the Analyze Particles command to obtain information about the pores, such as the area or Feret’s diameter (Figure 1).

### 2.4. Cell Adhesion Assay

**Figure 2:**
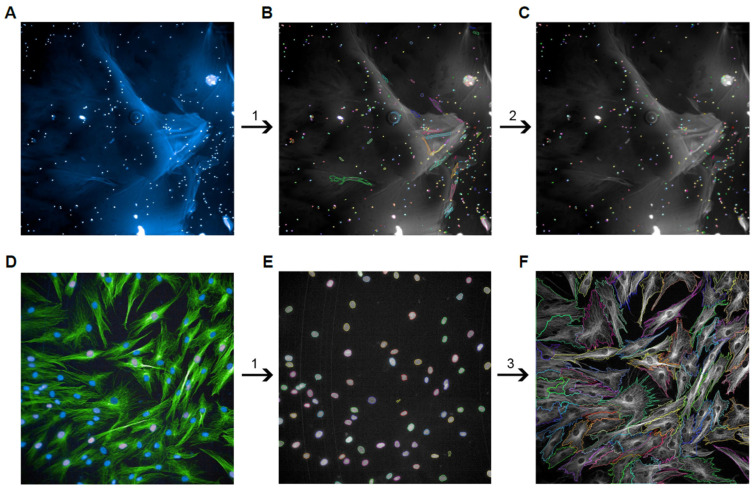
Image analysis sequence for nuclei quantification and cell morphology analysis. To calculate the number of nuclei adhered to Puramatrix and TCPs after different periods (cell adhesion assay), the images acquired at 5× magnification were analyzed as follows: the original image (**A**) was processed to (1) identify nuclei using pre-existing software methods (**B**). Next, (2) the area of the nuclei was calculated and those that did not have specific characteristics were excluded from the analysis (**C**). To calculate the area of the cells in the different culture conditions, the images acquired at 20× magnification were analyzed as follows: the original image (**D**) was processed to (1) identify nuclei using pre-existing software methods (**E**). Next, (3) the cytoplasm was selected using pre-existing software methods (**F**), followed by calculation to verify the size of the cell area.

Cell adhesion on PURA was evaluated following a previously described protocol [19]. The hASCs (5 × 10^3^ cells/well) were plated onto the PURA or directly in tissue culture plates (TCPs) and incubated for 20, 40, or 120 min at 37 °C and 5% CO_2_. Immediately after each incubation period, the plates were agitated at 100 rpm for 5 min. Then, the cells were washed using 1X PBS, fixed using 4% paraformaldehyde (PFA), and later stained with DAPI (4′,6-diamidino-2-phenylindole). DAPI was excited at a wavelength of 355–385 nm and its fluorescence emission was recorded at wavelengths around 430–500 nm. Quantification of the adhered cells was performed using an Operetta HTS imaging system (PerkinElmer, Waltham, MA, USA) at 5× magnification with 9 fields of view. This was followed by data analysis using the Harmony 4.9 software (PerkinElmer, Waltham, MA, USA). The analysis sequence for counting nuclei in the software was as follows: Input image (original image) (Figure 2A) >> Find nuclei (Figure 2B) >> Calculate morphology properties (nuclei area) >> Select population (based on nucleus area, to exclude image artifacts) >> Define results (number of objects; number of nuclei) (Figure 2C).

### 2.5. Cell Growth Curve

Cell growth dynamics on PURA were assessed via DNA extraction and quantification after 1, 5, and 20 days of cell culture. After each culturing period, DNA was extracted from the cells following a homebrew DNA extracting protocol using Proteinase K enzymatic digestion and quantified using a NanoDrop™ One Microvolume UV-Vis Spectrophotometer (Thermo Scientific™).

### 2.6. Proliferation Assay

For the assessment of cell proliferation, an EdU incorporation assay was performed. Suspensions of 1.5 × 10^3^ cells/well were plated on PURA or TCPs and cultured for 5 days. The EdU assay was performed following the manufacturer’s instructions (Click-iT™ EdU Cell Proliferation Kit for Imaging AlexaFluor™ 594, Invitrogen^®^ Carlsbad, CA, USA). The EdU incorporation analysis was performed using the Operetta HTS imaging system (PerkinElmer, Waltham, MA, USA) confocal module at 20× magnification with 9 fields of view, followed by data analysis using the Harmony 4.8 software (PerkinElmer, Waltham, MA, USA).

### 2.7. Cell Viability

The viability of cells on PURA was evaluated by quantifying lactate dehydrogenase enzyme (LDH) using the CytoTox 96^®^ Non-Radioactive Cytotoxicity Assay kit (Promega, Madison, WI, USA). The hASCs were cultured on the coating or TCP (1.5 × 10^3^ cells/well) and after 5 days the culture media was harvested and prepared for analysis according to the manufacturer’s protocol. LDH concentration was determined by measuring the absorbance of the resulting solutions at 490 nm using a Synergy H1 Hybrid Multiplate Microplate Reader (Biotek^®^, Winooski, VT, USA).

### 2.8. Cell Morphology

To assess alterations in hASC morphology, 1.5 × 10^3^ cells/well were plated on TCPs or PURA and cultured for 5 days. After this period, cells were fixed with 4% PFA and incubated with rabbit anti-β-Tubulin antibodies (ab15568, Abcam, Cambridge, UK diluted in 1% PBS/BSA (bovine serum albumin) and 0.5% Triton X-100, overnight at 4 °C under gentle agitation. The samples were washed thrice for 5 min with 1% BSA/PBS and 0.1% Triton X-100 solution. Then, anti-rabbit Alexa Fluor 488 (A21206; Invitrogen Life Technologies^®^, Carlsbad, CA, USA) secondary antibodies diluted with 1% PBS/BSA and 0.1% Triton X-100 solution were added to the samples. After incubation for 3 h, samples were washed again and stained with DAPI. Cell imaging was performed using the confocal microscope of an Operetta HTS imaging system (PerkinElmer, Waltham, MA, USA) at 20× magnification. Cell morphology analysis was performed using the Harmony 4.8 software (PerkinElmer, Waltham, MA, USA). The analysis sequence for calculating cell area (morphology analysis) in the software was as follows: Input image (original image) (Figure 2D) >> Find nuclei (Figure 2E) >> Select population (to remove the objects on image borders) >> Find cytoplasm (based on the methods available) (Figure 2F) >> Calculate morphology properties (area, width, length, and others) >> Define results (number of objects, cell area, and others). In addition, to better understand cell behavior on PURA, after hASC plating on the coating, images of the cell plates were taken every 5 days over a total of 20 days using Leica DM IL LED (Leica Microsystems, Wetzlar, Germany).

### 2.9. Immunophenotypic Profiling

The immunophenotypic profile of hASCs cultured in PURA was evaluated using flow cytometry. After 10 days of culturing in PURA, cells were trypsinized and resuspended in 1% PBS/BSA for 1 h at 4 °C. Subsequently, hASCs were centrifuged at 700× *g* for 5 min and resuspended in a 1% PBS/BSA solution containing the diluted antibodies. The following antibodies were used: FITC-conjugated anti-CD90 (E-Bioscience, Carlsbad, CA, USA); APC-conjugated anti-CD73 (E-Bioscience, Carlsbad, CA, USA); PE-conjugated anti-CD105 (E-Bioscience, Carlsbad, CA, USA); PE-conjugated anti-CD140b (BD Biosciences, San Diego, CA, USA); FITC-conjugated anti-CD34 (Miltenyi Biotec, Gaithersburg, MD, USA); APC-conjugated anti-CD45 (E-Bioscience, Carlsbad, CA, USA); APC-conjugated anti-HLA-DR (Invitrogen Life Technologies^®^, Carlsbad, CA, USA); FITC-conjugated anti-CD29 (E-Bioscience, Carlsbad, CA, USA); PE-conjugated anti-CD49e (E-Bioscience, Carlsbad, CA, USA); APC-conjugated anti-CD44 (BD Biosciences, San Diego, CA, USA); PE-conjugated anti-CD56 (BD Biosciences, San Diego, CA, USA); APC-conjugated anti-CD49c (BD Biosciences, San Diego, CA, USA), and PE-conjugated anti-CD166 (BD Biosciences, San Diego, CA, USA). The cells were incubated with the diluted antibodies for 1 h at 4 °C and then washed with 1X PBS. After another centrifugation, the cells were fixed with 4% PFA for 10 min, washed with 1X PBS, centrifuged, and resuspended in 1X PBS. Flow cytometry was performed in a FACSCanto II flow cytometer (BD Biosciences) and the data were analyzed using FlowJo software version 10.8.1.

### 2.10. Chondrogenic Differentiation

To evaluate the effects of PURA on the chondrogenic potential of hASCs, the cells were plated in 24-well plates (1.5 × 10^4^ cells/well) and maintained in culture for 3 days before the differentiation protocol. To assess whether cell confluence could influence chondrogenic differentiation on PURA, another treatment group (PURA2) was added where 7 × 10^4^ cells/well were plated. The hASCs were induced for chondrogenic differentiation for a period of 21 days using Human Mesenchymal Stem Cell (hMSC) Chondrogenic Differentiation Medium BulletKit 10TM (Lonza^®^, Walkersville, MD, USA; catalog number PT-3003). The differentiation medium was supplemented with 10 ng/mL of TGF-β3 (Lonza^®^, Walkersville, MD, USA; catalog number PT-4124) in each of the medium changes, which was performed every 3–4 days. After 21 days of culturing, the efficiency of chondrogenic differentiation was assessed using Safranin O staining, glycosaminoglycan (GAG) quantification, and expression of chondrogenic genes using real-time quantitative polymerase chain reaction (RT-qPCR).

### 2.11. Safranin O Staining

The induced (chondro) and non-induced (control) samples were initially fixed with 4% PFA, followed by three washes with 1X PBS. Then, cells were stained with 0.1% Safranin O solution (Safranin O diluted in deionized water) for 30 min (Sigma-Aldrich, St. Louis, MO, USA). After the removal of the Safranin O solution, plates were washed three times with 1X PBS to remove any excess dye. The plates were analyzed at 20× magnification under a DMi8 inverted fluorescence microscope (Leica Microsystems, Wetzlar, Germany).

### 2.12. GAG Quantification

The chondrogenic potential of differentiated hASCs on TCPs or PURA was evaluated by GAG quantification using the 1.9-dimethylmethylene blue (DMMB) dye-binding assay. After applying the differentiation protocol, cells were incubated in a papain digestion solution (100 mM sodium phosphate buffer, 10 mM NA2EDTA, 10 mM L-cysteine, and 0.125 mg/mL papain) for 18 h at 65 °C. Cells were then centrifuged at 10,000× *g* for 10 min and the supernatant was harvested for DNA and GAG quantification. The DNA was quantified using the Qubit™ dsDNA HS Assay kit (Molecular Probes; Invitrogen Life Technologies^®^, Carlsbad, CA, USA) following the manufacturer’s instructions. For GAG quantification, a solution of 0.16% DMMB (Sigma-Aldrich, St. Louis, MO, USA), 0.24% NaCl, 0.30% glycine, and 10 mM HCl was prepared and added to the supernatant containing extracted GAG. The absorbance of the resulting solutions was measured at 520 nm using a Synergy H1 Hybrid Multiplate Microplate Reader (Biotek^®^, Winooski, VT, USA). The GAG content was selected to fit within the range of a standard curve (sGAG reference standard; Biocolor, Northern Ireland, UK). The results were normalized using the previously quantified DNA to determine each sample’s GAG concentration.

### 2.13. Real-Time Polymerase Chain Reaction (RT-qPCR)

After 21 days of chondrogenic differentiation, RNA was isolated and cDNA synthesis and RT-qPCR were performed. RNA was extracted using TriReagent and the RNA isolation followed the Direct-zol™ RNA MiniPrep manufacturer’s instructions (Zymo Research). cDNA was subsequently synthesized using the ImProm-II™ Reverse Transcription System (Promega, Madison, WI, USA) kit instructions. RT-qPCR samples were prepared following the manufacturer’s instructions (GoTaq^®^ qPCR and RT-qPCR; Promega, Madison, WI, USA) and performed in technical triplicates from three differentiations. The analyzed genes were: COL2A1 (collagen type II; forward primer 5′-CATCCCACCCTCTCACAGTT-3′ and reverse primer 5′-GCCTCTGCCTTGACCCGAAG-3′), SOX9 (SRY-box transcription factor 9; forward primer 5′-AAGAACAAGCCGCACGTCAA-3′ and reverse primer 5′-CCGTTCTTCACCGACTTCCTC-3′), COL1A1 (collagen type I; forward primer 5′-AGGGCTCCAACGAGATCGAGATCCG-3′ and reverse primer 5′-TACAGGAAGCAGACAGGGCCAACGTCG-3′), ACAN (aggrecan; forward primer 5′-CACTGTTACCGCCACTTCCC-3′ and reverse primer 5′-GACGATGCTGCTCAGGTGTG-3′), MMP13 (matrix metallopeptidase 13; forward primer 5′-CATGAGTTCGGCCACTCCTT-3′ and reverse primer 5′-CCTCGGAGACTGGTAATGGC-3′), COL10A1 (collagen type X; forward primer 5′-CCAGCACGCAGAATCCATCTGA-3′ and reverse primer 5′- CTTGGTGTTGGGTAGTGGGC-3′), and RNApol2 (RNA polymerase II; forward primer 5′-TACCACGTCATCTCCTTTGATGGCT-3′ and reverse primer 5′-GTGCGGCTGCTTCCATAA-3′), which was used as a housekeeping gene. Data were obtained using a QuantStudio™ 5 Real-Time PCR System (Thermo Fisher, Waltham, MA, USA) and analyzed according to the 2-ΔΔCt method using QuantStudio™ Design and Analysis Software v1.5.2.

### 2.14. Statistical Analysis

Statistical analysis was performed using GraphPad Prism version 8.0. An unpaired student’s *t*-test was conducted to compare two groups, while an ordinary one-way ANOVA and Tukey’s post-test were applied to compare multiple treatment groups. All data are expressed as mean ± standard deviation (SD), within a confidence interval of 95%.

## 3. Results

### 3.1. Puramatrix™ Supports hASC Growth

**Figure 3:**
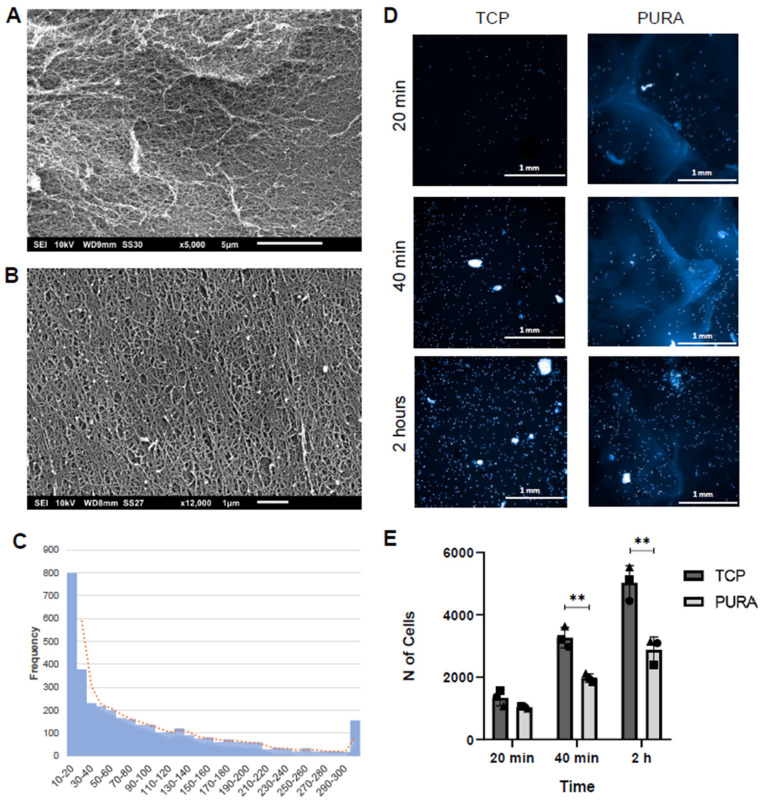
Puramatrix allows the adhesion of hASCs in a time-dependent manner. Characterization of Puramatrix™ topography using scanning electron microscopy: (**A**,**B**) view of the Puramatrix™ fibrous network. (**C**) Pore size (Feret’s diameter) distribution (nm). (**D**) Representative images of hASCs adhered to TCPs or PURA after 20 min, 40 min, and 2 h of incubation (DAPI was used to stain cell nuclei; scale bar = 1 mm). (**E**) Quantification of adhered cells after 20 min, 40 min, and 2 h of incubation. Each symbol (square, circle, and triangle) represents a different hASC donor. An unpaired student’s *t*-test was performed to compare the two treatment groups. ** *p* < 0.01.

The PURA nanotopography was characterized using SEM, as shown in Figure 3A,B. Further inspection of the hydrogel layer confirmed that PURA comprises a highly porous, compact nanofibrous array with pore diameters ranging from 10 to 200 nm (Figure 3B,C). The hydrogel layer showed no signs of damage or degradation after 5 and 10 days. Then, we evaluated whether PURA could affect hASC adhesion. After 20 min of incubation, both TCPs and PURA had the same number of cells adhered; however, as time progressed, both at 40 min and 2 h of incubation, PURA had about half the number of cells adhered in comparison to TCP (Figure 3D,E), showing that cells adhered slower to PURA compared to TCPs.

**Figure 4:**
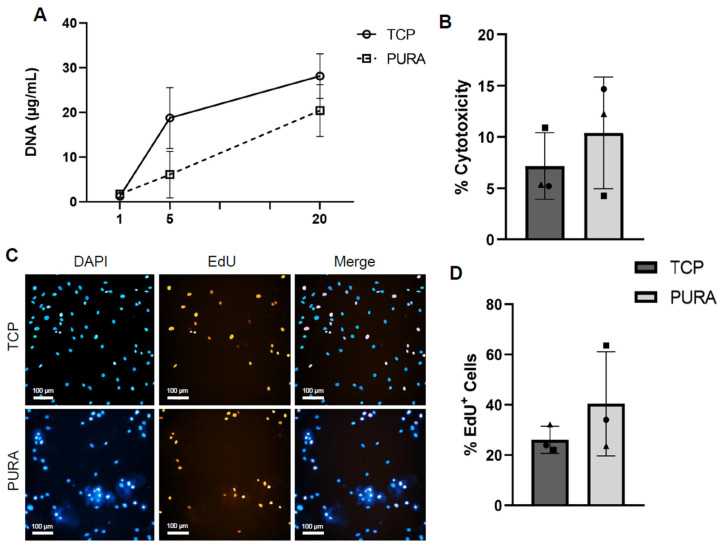
Biocompatibility of the Puramatrix™ coating. A growth dynamics assay was performed via DNA extraction and quantification, while an LDH assay was performed to assess cytotoxicity (viability) and an EdU incorporation assay was performed to evaluate cell proliferation. (**A**) Growth dynamics of cells in TCPs and PURA after 1, 5, and 20 days in cell culture. (**B**) Percentage of cytotoxicity in TCPs and PURA after 5 days in cell culture. (**C**) Representative images of the EdU (red labeling) incorporation assay after 5 days in cell culture (scale bar = 100 μm). (**D**) Quantitative analysis of the percentage of EdU+ cells. Each symbol (square, circle, and triangle) represents a different hASC donor. An unpaired student’s *t*-test was performed to compare the two treatment groups.

After adhesion, we assessed hASC growth dynamics on PURA. After 1 day of cell culture, DNA quantification indicated a similar number of cells in TCP and PURA cultures at this time point (Figure 4A). After 5 days, a marked increase in the amount of DNA was observed in the TCP culture compared to PURA; however, the effect size was small (TCP: 18.77 μg/mL ± 6.81; PURA: 6.1 μg/mL ± 5.21). At 20 days, both cultures had similar amounts of DNA (TCP: 28.13 μg/mL ± 4.99; PURA: 20.43 μg/mL ± 5.78) (Figure 4A). These results showed that hASCs cultured in PURA did not have as rapid a proliferation rate as those cultured in TCPs in the first five days, but a slow and steady growth throughout the 20-day experiment, showing that Puramatrix™ may modulate cell growth and maintenance.

Considering the differences in cell growth dynamics in PURA compared to TCPs—mainly after 5 days of culture—we investigated whether PURA affected the viability or proliferation capacity of hASCs. The PURA treatment had no cytotoxic effect on the cells (Figure 4B), as the percentages of cell death were similar between TCPs (7.15% ± 3.25) and PURA (10.39% ± 5.45). Representative images of the EdU assay showed that both culture conditions allow the proliferation of hASCs, which was confirmed after quantification (Figure 4C,D). Quantitative analysis revealed that Puramatrix™ did not affect cell proliferation, with the percentage of EdU-positive cells in PURA being comparable to that in TCPs on day 5 of culturing (TCP: 34.40% ± 10.06; PURA: 40.41 ± 20.70). Combined, these results suggest that the cell population undergoes adaptation to the Puramatrix™ substrate in the first 5 days of culturing.

### 3.2. Puramatrix™ Alters hASC Morphology but Not its Immunophenotype Profile

**Figure 5:**
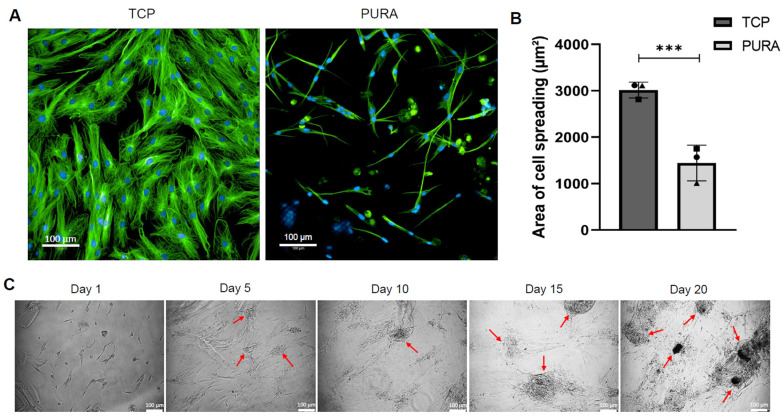
Characterization of cell morphology and cell aggregate formation on Puramatrix™. Cell morphology was characterized by β-Tubulin immunostaining (green labeling). (**A**) Representative images of cells in TCPs and PURA (scale bar = 100 μm). (**B**) Quantitative analysis of the area of cell spreading in TCPs and PURA. Each symbol (square, circle, and triangle) represents a different hASC donor. (**C**) Representative images of cells cultured in PURA for 1, 5, 10, 15, and 20 days showing aggregate formation throughout the culture period. Red arrows indicate the cell aggregates. An unpaired *t*-test was used to compare the two treatment groups. ***: *p* < 0.005.

One aspect that drew attention after 5 days in culture was the apparent change in cell morphology. When cells were stained with β-tubulin, it was observed that hASCs in PURA exhibited a variety of morphologies and that the interaction between the cells and the Puramatrix™ coating also resulted in the formation of cell aggregates (Figure 5A). Quantitative analysis of the cell-spreading area showed that cells that adhered to PURA were half the size of cells on TCPs (Figure 5B). Considering the importance of cell aggregation in the initial stages of cartilage formation, we further investigate the establishment of these aggregates on the Puramatrix™. The images acquired over 20 days of culturing showed hASC growth and formed cell aggregates in PURA, with an increase in cell confluence and the number and size of aggregates throughout the experiment (Figure 5C).

**Figure 6:**
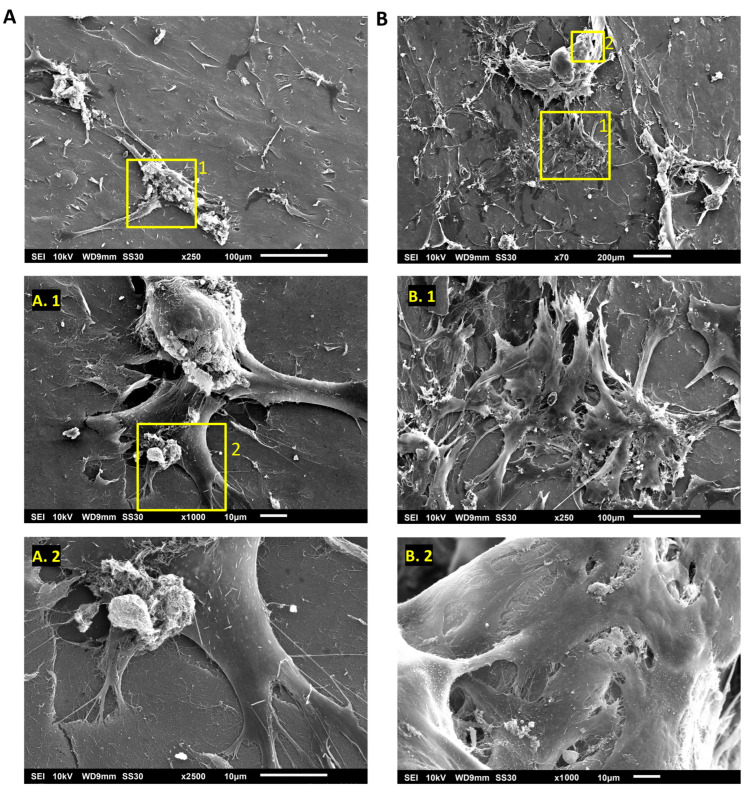
Ultrastructural characterization of cell morphology on Puramatrix™. (**A**) Cells adhered to PURA after 5 days in cell culture. (**A.1**) Cell aggregates interacting with the Puramatrix™ coating. (**A.2**) Cell cytoplasmic protrusions. (**B**) Cells adhered to PURA after 10 days in cell culture. (**B.1**) Cell aggregates stacked on top of each other. (**B.2**) Cell aggregates interacting with each other and with the Puramatrix™ substrate.

To better characterize the morphology of hASCs adhered to the Puramatrix™ and investigate the aggregates, we performed SEM analysis after 5 and 10 days of cell culturing. As shown in Figure 6, after 5 days in cell culture, PURA treatment exhibited low cell confluence, a variety of cell morphologies, and some amorphous structures (Figure 6A). Further inspection of these structures revealed that they are indeed cell aggregates, with cells interacting with each other and with the coating surface (Figure 6A). Confluence was higher after 10 days in culture, with larger aggregates composed of cells and hydrogel (Figure 6B). The cell aggregates were also packed on top of each other (Figure 6B).

Next, we checked whether changes in morphology could cause changes in the hASC immunophenotypic profile. After 10 days in PURA, hASCs showed no expressive changes in the percentage of positive cells for hASC classic surface markers such as CD90, CD73, CD105, CD140b, CD34, and CD45 (Table 1). However, almost 17% of cells cultured in PURA expressed HLA-DR, an antigen that is normally negative in MSCs, on their surfaces. The expression of other adhesion molecules such as CD29, CD49e, CD44, CD56, CD49c, and CD166 was also measured, but no changes in the percentage of cells expressing these molecules were found in comparison with cells in TCPs (Table 1). Thus, despite the change in morphology, hASCs maintain the same expression of surface markers.

### 3.3. Puramatrix Affects Chondrogenic Differentiation of hASCs

Based on the observed cell culture dynamics in PURA, including morphological differences and induction of cell aggregation, we investigated the potential influence of Puramatrix™ on the induction of chondrogenic differentiation. We decided to include a new experimental group (PURA2) with a higher number of cells given the previous results (Figure 4A, Figure 5A, and Figure 6) and the importance of confluence in cell differentiation.

**Figure 7:**
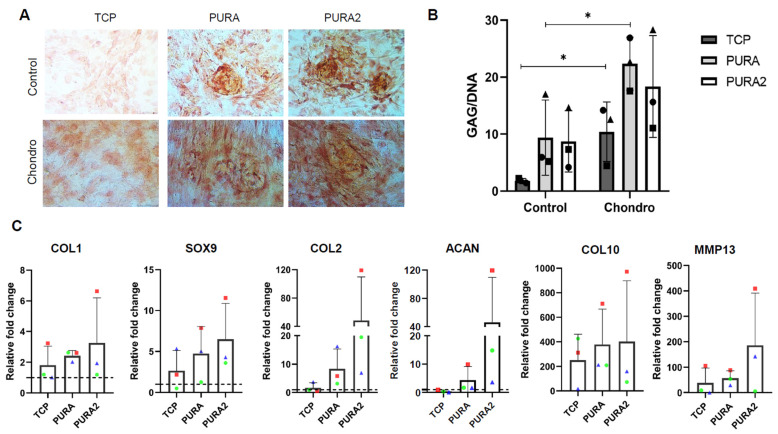
Chondrogenic differentiation of hASCs in Puramatrix™. (**A**) Safranin O staining (in Red) of hASCs induced (Chondro) and not induced (Control) to undergo chondrogenic differentiation. (**B**) Quantification of GAG deposition after chondrogenic induction. (**C**) mRNA expression levels of COL1, SOX9, COL2, ACAN, COL10, and MMP13 after chondrogenic induction. Data are represented as mean ± SD and were compared to the non-induced group (Control). Each symbol (square, circle, and triangle) represents a different hASC donor. One-way ANOVA was performed to compare the treatment groups. * *p* < 0.05.

After cell differentiation induction, Safranin O staining was performed to qualitatively assess the deposition of sulfated GAGs. hASCs cultured in TCPs in expansion media (control) were weakly stained (Figure 7A), while cell aggregates present in PURA and PURA2 appeared a little more stained. Under chondrogenic induction, the cell aggregates present in both PURA and PURA2 were highly stained—more than in TCP—indicating significant production and accumulation of sulfated GAGs (Figure 7A). The quantification of GAG production revealed a tendency of higher GAG deposition by cells cultured on PURA and PURA2 in comparison to those cultured in TCPs (Figure 7B), both by cells in expansion media (control) and chondrogenic media (chondro).

Chondrogenesis was further quantitatively examined using RT-qPCR to analyze the expression profiles of genes associated with chondrogenic differentiation. Similar to GAG quantification, we did not observe significant differences between conditions (Figure 7C). However, it is noted that the COL2 gene, for example, was expressed on average 50 times more in chondro x control in PURA2, and an average of 10 times more in PURA, while in TCP it was only expressed 2 times more in chondro than control (Figure 7C). This suggests that Puramatrix™ may induce a higher expression of chondrogenic genes.

## 4. Discussion

Puramatrix™ is a commercially available self-assembling peptide hydrogel that possesses remarkable characteristics that make it highly suitable for cartilage tissue engineering. Its biocompatibility and ability to create a three-dimensional ECM environment closely resembling natural cartilage make it an attractive candidate for regenerative applications, including cartilage tissue engineering research [13].

Initially, we characterized the microtopography of the Puramatrix™ and conducted comprehensive biocompatibility assays to evaluate the response of hASCs to this culturing condition. Several factors can affect cellular behavior when cells are cultured in hydrogels, including composition, stiffness, pore size, degradation, and viscoelasticity [20]. The coating used in this study had nanopores between 10 and 200 nm. Previous studies have already demonstrated that alumina membranes with pore diameters of 20 nm allow more adhesion of macrophages but less cytokine release compared to alumina membranes with pore diameters of 200 nm [21]. A comparison of TiO2 nanotubes of 20, 50, or 100 nm pore diameters showed that there was higher hMSC osteogenesis induction in nanotubes with 20 nm pore diameters, while the human osteoblast lineage preferred the 50 nm nanotubular structures for osteoblastic maturation [22]. In addition, MSCs encapsulated in a hydrogel with ~5 nm pore size affect the secretome composition compared to plating cells in a scaffold with a ~120 µm pore size [23]. It is worth noting that pore size changes the cell behavior in different aspects, but the composition of the hydrogel, the culture methodology (seeding or encapsulation) as well as other factors also contribute to the observed effect. Furthermore, smaller pores allow for a controlled release of growth factors such as TGFβ [24]. Considering the potential of Puramatrix™ coating, future perspectives include encapsulating the hASCs in Puramatrix™ and functionalizing this hydrogel with growth factors (such as TGF β) that can allow its steady release, offering a lasting stimulus to the cells.

Moreover, previous atomic force microscopic analysis by Ortineau and colleagues [25] demonstrated that different concentrations of Puramatrix™ hydrogel exhibit distinct topographic features. At concentrations of 0.15% and 0.25%, the hydrogel formed a network of thin and scattered fibers. However, the fibrous network became denser at a concentration of 0.5%, indicating a more compact structure [25]. Additionally, increasing the concentration of Puramatrix™ hydrogel not only reduced the size of the hydrogel pores but also increased its stiffness [26], potentially influencing cell adhesion, proliferation, and migration [27]. Our SEM analysis confirmed that 0.5% Puramatrix™ resulted in a compact nanofibrous network. Despite its compact structure, Sharma and coworkers [28] demonstrated that Puramatrix™, at a concentration of 0.5%, allowed MSCs to effectively migrate into the matrix, avoiding the stress caused by the encapsulation process [28]. In another study conducted by Shivachar [29], this concentration of Puramatrix™ provided a structure with pore sizes large enough for neurons to extend their dendrites and form synapses with neighboring cells. However, although the pore size increased at lower concentrations (<0.25%), the hydrogel was too soft and fragile to handle [29]. This issue was avoided in our study, as the SEM analysis revealed that Puramatrix™ at 0.5% concentration remained resistant to culture media exchanges for a period of up to 10 days, meaning it could offer cells a lasting stimulus throughout the cell culture process. This hydrogel structure allowed the adhesion of hASCs but at a slower pace compared to the tissue culture plate. While 2 h was enough for all the plated cells to adhere to the TCP, cells in PURA needed more time to fully interact with the hydrogel layer. This was confirmed during the growth dynamics assay since the number of cells in TCPs and PURA were similar following the first 24 h of cell culturing. The growth dynamics assay also revealed that the Puramatrix™ hydrogel may modulate cell proliferation and maintenance. While hASCs in PURA grew slowly and steadily throughout the experiment, cells in TCPs showed rapid growth during the first 5 days and steady growth after that. One possibility is that hASCs in TCPs may be simply reaching a higher confluence earlier, as seen in Figure 5A, which can end up reducing the cell’s proliferation rate after the cells reach the physical limits of the area of the plate well [30].

The observed difference in growth dynamics in our study may be attributed to changes in cell death or proliferation rates. However, our investigation demonstrated that this was not necessarily the case. We found that Puramatrix™ hydrogel was not cytotoxic and did not increase the percentage of proliferating hASCs, which suggests that factors other than cell death or proliferation mechanisms may underlie the disparities in the observed growth patterns. Although it remains unclear what caused these differences, taking note from our microscopy analysis (adhesion assay and SEM), we hypothesize that cells on PURA require a longer time to settle in this new environment. While cells on TCPs are already actively dividing, cells on Puramatrix™ hydrogel undergo an initial adaptation period before entering the proliferative stage. In physiologically relevant ECMs, such as Puramatrix™, cells often exhibit distinct migration and proliferation patterns [31]. The need for matrix remodeling [32] and the mechanical cues triggered by the softer substrate [33] may be exactly what caused the differences in cell behavior and growth dynamics seen in our study. Overall, our findings are consistent with previous studies that have employed various cytotoxicity and cell interaction assays to demonstrate Puramatrix™ biocompatibility, such as the diffusion assay in agar (ISO 10993-5) and the human plasma assay (ISO 10993-4) [27]. For instance, Cavalcanti et al. [34] conducted a study utilizing dental pulp stem cells encapsulated in Puramatrix™ hydrogel, testing different cell densities and Puramatrix™ concentrations (ranging from 0.05% to 0.25%). Their results demonstrated that cells were able to proliferate within 72 h, and no differences were observed among the various cell densities and concentrations [34]. Although our Puramatrix™ concentration was higher (0.5%), we confirmed that cells remained viable in the Puramatrix hydrogel even at higher concentrations.

The impact of Puramatrix™ on hASC morphology was remarkable. This could potentially be attributed to the soft nature of Puramatrix™ hydrogel, as it is widely known that substrate stiffness plays a crucial role in regulating cell behaviors such as spreading [35]. A study by Sun and colleagues [36] delved into the influence of ECM stiffness on cell morphology by investigating MCF-7 cells cultured on collagen-coated polyacrylamide (PAAm) gels with varying elastic moduli. The results revealed that cells that adhered to the softer surface had a rounded morphology and smaller spreading area. In contrast, cells that adhered to the stiffer surface displayed a spindle shape and a larger spreading area [36]. Interestingly, in the current study, when the hASCs were cultured on Puramatrix hydrogel, a soft substrate, they did not exhibit a uniform round shape. Instead, they displayed a variety of morphologies and cell aggregation, as observed through fluorescence microscopy and SEM analysis, ultimately resulting in a smaller cell spreading area.

In relation to cell aggregation, Ortinau and colleagues [25] previously reported the formation of aggregates in human neural progenitor cells in Puramatrix™ in concentrations of 0.25 and 0.5%, with more compact spheroids being formed at the higher concentration. In our study, it was observed that the formation of cell aggregates on PURA occurred spontaneously over the course of the 20-day culture period, as the aggregates gradually increased in both quantity and size. SEM analysis revealed that the aggregates were formed both by cells interacting with the substrate and cells interacting with each other, showing that Puramatrix™ hydrogel did not only influence the morphology of the hASCs but also induced the aggregation of these cells, leading to the formation of high-density cellular structures. These findings hold significant promise for cartilage tissue engineering, as cell aggregation is a critical step in the chondrogenic differentiation process [37]. Although the morphology of the hASCs was affected by Puramatrix™, the immunophenotypic profile of the cells was not significantly impacted under the given culture conditions, at least for the markers evaluated in this study.

Building upon these previous observations, we investigated the potential of the Puramatrix™ hydrogel to promote chondrogenesis. The assessment of chondrogenic differentiation through Safranin O staining and quantification of GAG production revealed a noteworthy trend suggesting that Puramatrix™ hydrogel may have an inherent capability to induce GAG production. Moreover, it had an augmentative effect on the potential of hASCs to produce this ECM component spontaneously and upon induction of chondrogenic differentiation. These findings imply a potential synergistic interaction between the Puramatrix™ and the chemical inducers present in the chondrogenic differentiation medium. The findings obtained in our study align with previous research conducted by Kopesky et al. [15], supporting the notion that Puramatrix™ hydrogel possesses the remarkable capacity to induce glycosaminoglycan (GAG) production during chondrogenic differentiation. In their study, it was demonstrated that bovine MSCs in Puramatrix™ hydrogel had twice the level of GAG production compared to those in an agarose hydrogel. Additionally, Taufer et al. [16] conducted a study on trunk neural crest cells from quail embryos undergoing chondrogenic differentiation in 0.15%, 0.25%, and 0.5% Puramatrix™. Their findings, though a different experimental model, corroborate our observations, as they detected aggregates containing cartilage extracellular matrix [16]. Collectively, these findings highlight the remarkable potential of Puramatrix™ hydrogel in inducing GAG production during chondrogenic differentiation.

Furthermore, our gene expression analysis provides further evidence supporting the potential for PURA to promote chondrogenic differentiation. The expression of chondrogenic genes such as SOX9, COL2, and ACAN was upregulated in cells cultured with Puramatrix™ hydrogel. These findings align with the observations made by Betriu and E. Semino [38], who reported a twofold increase in SOX9 expression in MSCs cultured in 0.3% Puramatrix™ compared to tissue culture plates. While these results are promising, it is important to consider that an increase in the expression of hypertrophic genes such as COL10 and MMP13 was also observed in our study [36]. This may suggest the potential of the Puramatrix™ hydrogel to induce chondrocyte hypertrophy. Further investigations are needed to explore whether this differentiation influence exerted by PURA occurs under different conditions, such as in vivo environments. On the other hand, it is worth noting that signs of hypertrophic chondrogenic differentiation are commonly observed in cartilage tissue engineering research. For instance, a recent study showed that MSCs cultured in hydrogels composed of gelatin methacryloyl (GelMA) and different concentrations of methacrylated chondroitin sulfate (CSMA) increased COL10 expression across all concentrations [39]. These findings highlight the challenge of hypertrophic differentiation and emphasize the need to investigate strategies aimed at minimizing or inhibiting this process.

A limitation encountered throughout our study relates to the variability observed across biological replicates. This variability can be attributed to inherent differences among hASC donors, leading to variations in the chondrogenic differentiation potential of each biological replicate. This was also described for other MSCs, such as bone-marrow MSCs, during chondrogenic differentiation [40]. While increasing the number of donors could potentially mitigate this limitation, it is important to acknowledge that the need to consider variations in donor chondrogenic potential has already been highlighted in a study by Kim et al. [41]. In their research, they observed significant differences in the functional chondrogenesis of hMSCs within hyaluronic acid hydrogels across eight different donors. Hence, for clinical applications, it may be necessary to tailor treatment settings to a per-donor basis to achieve more robust cartilage tissue formation [36]. Furthermore, it is important to acknowledge that the results presented in our study could be masked if the assays are solely performed using a single-cell lineage. This underscores the significance of employing a comprehensive methodology that encompasses multiple cell donors in tissue engineering investigations. By doing so, we can obtain a more comprehensive understanding of the potential applications and limitations of the proposed approaches in developing functional cartilage tissue constructs.

## 5. Conclusions

In this study, we were able to better understand how the SAPH Puramatrix™ influences hASCs. We noticed that PURA stimulated a considerable increase in the expression of chondrogenic genes and the production of GAGs that were more evident in some individual hASC biological replicates. In addition, an interesting and unexpected fact observed was the spontaneous formation of cellular aggregates in the Puramatrix™, one of the initial stages of chondrogenesis. Considering the induced shift in cell behavior and the observation of initial chondrogenesis induction, we postulated that Puramatrix™ can be used as a biomaterial for application in cartilage regeneration, although more studies are needed to confirm this. Optimizing a chondrogenic differentiation strategy in a donor-specific manner, assaying new concentrations of hydrogel and other growth factors, evaluating the secretome of MSCs cultured on PURA, and performing in vivo assays are interesting perspectives that can be pursued to expand our knowledge on Puramatrix™ and hASCs for cartilage tissue engineering.

## Figures and Tables

**Table 1:** Immunophenotypic profile of cultured hASCs in TCPs and PURA showing the percentage of each surface marker after 10 days of cultivation.

	TCPs	PURA
Antibodies	%	%
CD90	90.6 ± 8.78	83.0 ± 2.87
CD73	100.0 ± 0.00	95.0 ± 2.55 *
CD105	98.5 ± 0.98	98.5 ± 5.06
CD140b	98.4 ± 2.12	91.1 ± 7.28
CD34	20.1 ± 11.76	30.0 ± 1.90
CD45	-	-
HLA-DR	-	16.9 ± 2.75 ***
CD29	99.5 ± 0.35	94.8 ± 2.4 *
CD49e	100.0 ± 0.00	95.6 ± 4.03
CD44	100.0 ± 0.00	100.0 ± 0.00

Unpaired *t*-test. * *p* = 0.05; *** *p* = 0.005 compared to TCPs.

## Data Availability

Data are contained within the article.
